# Evolutionary Diversification and Adaptive Evolution Analysis of the Plant HD-Zip IV Subfamily

**DOI:** 10.3390/genes16111348

**Published:** 2025-11-08

**Authors:** Yujun Li, Zhao Liu, Ghulam Qanmber, Le Liu, Huiyun Shi, Yuling Guo, Mengli Yu, Ghulam Hussain, Fanjia Peng, Kai Zheng, Fuguang Li

**Affiliations:** 1 Key Laboratory of Crop Genetic Improvement and Germplasm Innovation, College of Agronomy, Xinjiang Agricultural University, Urumqi 830052, China; 15974267467@163.com; 2 State Key Laboratory of Cotton Bio-Breeding and Integrated Utilization, Institute of Cotton Research, Chinese Academy of Agricultural Sciences, Anyang 455099, China; 3 Cotton and Sericultura Research Institute, Hunan Academy of Agricultural Sciences, Changsha 410125, China; 4 State Key Laboratory of Cotton Bio-breeding and Integrated Utilization, School of Agricultural Sciences, Zhengzhou University, Zhengzhou 450001, China

**Keywords:** gene, evolution, positive selection, terrestrialization, functional differentiation

## Abstract

**Background/Objectives:** The terrestrialization of plants has been a significant driver of plant evolution over hundreds of millions of years, and epidermal development plays a crucial role in adapting to terrestrial environments. It is therefore of great interest that the HD-Zip IV subfamily serves as a key regulator of epidermal and cuticle development in plants. However, research on their expansion trajectory, adaptive evolution, and functional divergence remains scarce. **Methods:** We conducted a functional divergence and adaptive evolution analysis in the plant HD-Zip IV subfamily and confirmed the functional differentiation caused by positive selection site mutations through binding affinity prediction analysis and EMSA experiments. **Results**: Our findings revealed that the HD-Zip IV subfamily has diversified into five distinct branches, with progressive expansion throughout plant evolution. These variations in the HD (homeodomain) and START drive the functional differentiation among the evolutionary branch, in particular, a distinct leucine-rich motif in HD and lipid-binding pockets. Furthermore, we identified several amino acid sites within the START domain that have been under selective pressure during plant evolution, as well as convergent evolutionary sites shared between early land plants and seed plants. **Conclusions**: Our findings show that the HD-Zip IV subfamily experienced a significant expansion in gene number and diversification of evolutionary branch from streptophyte algae to seed plants. During plant evolution, genomic duplication events and variations in the HD and START have contributed to its expansion in gene number and diversification, respectively. We suggest that the balanced coexistence of functional robustness and relaxed constraints in the HD-Zip IV subfamily may have underpinned its successful response to the challenges of land environment.

## 1. Introduction

As sessile organisms, plants have evolved unique adaptive strategies to cope with various abiotic and biotic stresses, such as drought tolerance, diverse reproductive models, and specialized pollination systems. The formation of these adaptive strategies primarily relies on a complex regulatory network mediated by plant-specific transcription factors [1,2]. The expansion of these transcription factors has been a critical driver of plant adaptive evolution [3]. Among them, the HD-Zip (homeodomain-leucine zipper protein) gene family plays a pivotal role in stress responses, epidermal cell specialization, and the synthesis of essential metabolites, all of which are indispensable for plant adaptation to terrestrial environments. In plant evolution, the HD-Zip family has progressively diverged into four branches: HD-Zip I, HD-Zip II, HD-Zip III, and HD-Zip IV [4]. Differences in gene structure, domain composition, and physiological functions characterize these branches.

Within its four branches, the HD-Zip IV subfamily characterized by the unique combination of a homeodomain (HD) and a START (steroidogenic acute regulatory protein-related lipid transfer) domain, which confers lipid/sterol-binding capacity and is essential for lipid metabolism and epidermal cell specialization [5,6]. Phylogenetic analyses reveal that HD-Zip IV genes originated in charophycean green algae and expanded substantially during plant terrestrialization, with gene duplication events occurring independently in bryophytes, lycophytes, and euphyllophytes [7,8]. This evolutionary expansion represents a primary form of START domain diversification in plants and coincides with major evolutionary transitions [9]. The HD expansion, in particular, has been proposed as a key evolutionary force in major events, such as the Cambrian explosion [10,11]. The expansion of the HD-Zip IV subfamily is essential for plant adaptation to terrestrial environments, including protection against drought, UV radiation, and high temperatures. Its members play critical roles in lipid synthesis, water status regulation, defense, and reproductive strategies [12,13]. In rice, the HD-Zip IV gene *ROC1* (*ROC1KO*) regulates leaf rolling and drought response by forming heterodimers with *ROC5* and *ROC8*, it has been experimentally validated that co-overexpression of *ROC1*/*ROC8* both enhanced drought tolerance to plants [14]. Another HD-Zip IV gene in rice, *ROC4*, has been identified as a positive regulator of wax biosynthesis and consequently enhances drought tolerance [15]. In *Arabidopsis. thaliana*, the HD-Zip IV gene *EDT1* (*Enhanced Drought Tolerance 1*) has been demonstrated to play a critical role in regulating root system architecture and leaf stomatal density. Furthermore, overexpression of *EDT1* leads to significantly enhanced drought tolerance in transgenic plants [16]. Across diverse crop species, HD-Zip IV genes have evolved specialized functions in regulating epidermal development, stress responses, and metabolic processes [17,18,19]. For example, in maize, the HD-Zip IV subfamily gene *OCL4* inhibits trichome development and regulates anther cell wall division, with over-expression (OE) plants exhibiting a macrohair phenotype [20]. In tomatoes, three HD-Zip IV subfamily genes promote interlocking trichome formation, supporting the cleistogamy reproductive mechanism [21]. In *Artemisia annua*, the HD-ZIP IV subfamily transcription factor *AaHD8* regulates cuticle-related enzyme genes, influencing leaf cuticle development [22]. These specialized adaptations demonstrate how HD-Zip IV genes enhance plant tolerance to environmental stresses by orchestrating the development of epidermal structures and their appendage synthesis, including leaf stomata, wax biosynthesis, and the cuticular layer [23]. The functional diversification of HD-Zip IV genes across plant lineages reflects their continuous evolution and adaptation. These transcription factors integrate metabolic signals through their START domains while driving epidermal cell fate determination, ultimately enabling plants to develop specialized epidermal features and produce protective compounds such as waxes, alkaloids, and resins to combat diverse biotic and abiotic challenges [24]. The HD-Zip IV subfamily members synergistically shape epidermal development and drive adaptive evolution in terrestrial plants [25].

Without a doubt, the evolution of the HD-Zip IV subfamily provides critical insights into the roles of lipid/sterol signaling and epidermal cell development in the plant terrestrialization process. Despite its evolutionary significance, research on HD-Zip IV subfamily genes has primarily focused on single gene functions, with limited attention to how they expand and undergo functional differentiation and whether their expansion follows any universal principles. To address these gaps, we selected 35 representative species spanning the major evolutionary lineages of plants for evolutionary analysis. A comparative analysis was conducted to trace the expansion trajectories of the HD-Zip IV subfamily during plant evolution. Adaptive selection and convergent evolutionary sites within the HD-Zip IV subfamily I branch were investigated. Furthermore, EMSA analysis confirmed that the L43R mutation in AtPDF2 significantly enhanced its binding specificity capacity to the tandem repeats of the L1 box core motif. This study aims at elucidating the molecular basis of functional differentiation and adaptive evolution within the HD-Zip IV subfamily, and analyzing the dynamics of its expansion during plant evolution. This study provides new insights into the adaptive evolutionary mechanisms driven by the HD-Zip IV subfamily and future functional research.

## 2. Materials and Methods

### 2.1. Preparation of Plant Species Genomic Data

In this study, genomic, protein sequence, and corresponding CDS data for 35 species were obtained from the Phytozome 13 database (https://phytozome-next.jgi.doe.gov/ (accessed on 11 September 2024)). These species were *Amborella trichopoda*, *Ananas comosus*, *A. thaliana*, *Aquilegia coerulea*, *Beta vulgaris*, *Brachypodium distachyon*, *Brassica rapa*, *Chara braunii*, *Chenopodium quinoa*, *Chrysanthemum indicum*, *Cinnamomum kanehirae*, *Cucumis sativus*, *Cycas panzhihuaensis*, *Elaeis guineensis*, *Ginkgo biloba*, *Glycine max*, *Gossypium hirsutum*, *Klebsormidium nitens*, *Litchi chinensis*, *Musa acuminata*, *Nelumbo nucifera*, *Nymphaea colorata*, *Oryza sativa*, *Physcomitrium patens*, *Populus trichocarpa*, *Rhododendron delavayi*, *Santalum yasi*, *Selaginella moellendorffii*, *Solanum lycopersicum*, *Solanum tuberosum*, *Spirodela coerulea*, *Spirogyra pratensis*, *Theobroma cacao*, *Vitis vinifera* and *Zea mays.* Additionally, genomic and protein sequences of 11 bryophytes and 10 lycophytes and ferns were retrieved from the National Genomics Data Centre (https://ngdc.cncb.ac.cn/ (accessed on 27 August 2024)). The species were *Adiantum capillus*, *Alsophila spinulosa*, *Azolla filiculoides*, *Bryum argenteum*, *Calohypnum plumiforme*, *Ceratodon purpureus*, *Ceratopteris richardii*, *Entodon seductrix*, *Fontinalis antipyretica*, *Hypnum curvifolium*, *Isoetes taiwanensis*, *Marchantia paleacea*, *Marchantia polymorpha*, *Marsilea vestita*, *Physcomitrium patens*, *Pohlia nutans*, *Salvinia cucullata*, *Selaginella lepidophylla*, *Selaginella moellendorffii*, *Selaginella tamariscina*, *Syntrichia caninervis*.

### 2.2. Time-Calibrated Phylogeny of 35 Plant Species and Whole-Genome Identification of HD-Zip IV Subfamily Genes

To identify homologous sequences of HD-Zip IV subfamily genes, homologous sequences of the HD-Zip IV subfamily were identified through a comprehensive search strategy. First, protein sequences of 16 known *A. thaliana* HD-Zip IV genes were used as queries to perform a local BLASTP search against the proteome datasets of the target species using TBtools v2.070 with E-value cutoff of 1 × 10^−10^. Second, to capture more divergent homologs that might have been missed by the similarity search, all candidate sequences were subjected to a domain-based verification using the HMMER module within TBtools. This step utilized the curated PFAM profiles for the Homeobox domain (PF00046.24) and the HD-Zip C-terminal domain (PF01852.14) [26]. Finally, to ensure a non-redundant and high-quality dataset, we removed fragmentary sequences (length < 300 amino acids) and retained only the longest protein isoform for each gene locus. The time-calibrated phylogeny for 35 selected plant species, and the time-calibrated phylogeny for 21 bryophytes, lycophytes and ferns, were constructed using TIMETREE 5 (https://timetree.org/ (accessed on 13 August 2024)). This resource synthesizes divergence time estimates from numerous published studies to generate a consensus timeline of evolution. The resulting trees, which use median divergence times for branch lengths, were visualized using TreeView V2.2.0 software. Simplified species illustrations for the phylogenetic tree were sourced from PhyloPic (https://www.phylopic.org/ (accessed on 23 September 2024)) [27].

### 2.3. Sequence Alignment and Phylogenetic Evolutionary Analysis

Identified HD-Zip IV subfamily gene sequences were aligned using the CLUSTALW module of MEGA 12, with the following parameters: Gap Opening Penalty setx sadscc to 10.00, Gap Extension Penalty to 0.2, and Delay Divergent Cutoff to 30% [28]. The phylogenetic tree was constructed using the Maximum Likelihood (ML) method in MEGA 12 with 2000 bootstrap replicates. Branches with bootstrap values ≥ 70% were considered moderately supported, and values ≥ 95% were considered highly supported. The final tree was visualized and annotated using Tree Of Life (iTOL) v5 [29].

### 2.4. Protein–DNA Docking and Visualization of the HD-Zip IV Subfamily

Protein structure prediction for conserved HD-Zip IV subfamily protein or HD was performed using AlphaFold2 [30]. The model with the highest pLDDT (Predicted Local Distance Difference Test) confidence score was selected for subsequent analysis. Protein–DNA docking analysis, focusing on interactions between HD and the L1-box core sequence (5′-TAAATG(C/T)A-3′), was conducted using the HDOCK server (hdock.phys.hust.edu.cn/ (accessed on 16 August 2024)). The docking search was performed in “Protein-DNA” mode, with the search space and sampling parameters set to default. The top 3 docking poses were generated and evaluated. Visualization and analysis of the 2D interaction diagrams of the DNA–protein complexes were carried out using LigPlot+. The 3D structures of the complexes were rendered and visualized using PyMOL Molecular Graphics System, Version 2.5.0 [31].

### 2.5. Amino Acid Conservation Analysis of HD in the HD-ZIP IV Subfamily

Amino acid conservation in HD of various HD-ZIP IV subfamily branches was analyzed using the ConSurf web server (https://consurf.tau.ac.il/ (accessed on 8 November 2024)) [32]. For each distinct branch, the member sequence that was closest in genetic distance to the branch’s consensus was selected as the template for that analysis. Multiple sequence alignments (MSA) were generated using MAFFT-L-INS-i against the UniRef90 sequence database. Conservation scores were calculated using the Bayesian method with the JTT evolutionary substitution model. The resulting conservation grades, ranging from 1 (variable) to 9 (highly conserved), were mapped onto the corresponding protein structure.

### 2.6. DAP-Seq Target Gene Profiling and Comparison Among Arabidopsis HD-Zip IV Transcription Factors

DAP-seq data for *Arabidopsis* HD-Zip IV subfamily genes (*PDF2*, *HDG1*, and *ANL2*) were retrieved from the Plant Cistrome Database (neomorph.salk.edu/dap_web/pages/index.php (accessed on 11 July 2024)) [33]. Gene functional enrichment analysis was performed using clusterProfiler R package, and data visualization was carried out with Origin 2021 software for data visualization and the KEGG database (kegg.jp/) for pathway analysis [34].

### 2.7. DNA–Protein Complex Binding Affinity Analysis

PDA-Pred software (https://web.iitm.ac.in/bioinfo2/pdapred/ (accessed on 6 November 2024)) was used to predict binding affinities between DNA and proteins [35]. Predictions were generated using the software’s default Random Forest regression model. The predictive performance of the model achieved a correlation coefficient of 0.86 and a mean absolute error (MAE) of 0.76 kcal/mol, indicating high reliability.

### 2.8. Functional Divergence and Adaptive Evolution Analysis

Functional divergence among 393 HD-Zip IV subfamily proteins was assessed using DIVERGE version 4, examining type I (Q1) and type II (Q2) divergence [36]. Adaptive evolution of 122 branch I HD-Zip IV subfamily genes was analyzed using EasyCodeML 1.4 under pre-set models, including pairwise comparisons (M0 vs. M3, M1a vs. M2a, M7 vs. M8, and M8a vs. M8) [37].

### 2.9. Electrophoretic Mobility Shift Assays

The coding sequences of AtPDF2 and AtPDF2-HD(L43R) were inserted into the pET-30a vector. AtPDF2-his and AtPDF2-HD_(L43R)_-his proteins were expressed in *E. coli* BL21 (DE3) by inducing with 0.5 mm IPTG for 12 h at 16 °C. The his-tagged proteins were purified using IDA-Ni agarose magnetic beads from Beyotime Biotechnology company (Beijing, China). The biotinylated probe of the TAATTAATTAATTAATTAATTAATTAATTAAT sequence and TCCTTCCTTCCTTCCTTCCTTCCTTCCTTCCT sequence was synthesized by Sangon Biotech (Shanghai, China). Electrophoretic mobility shift assays was performed using a chemiluminescence-based EMSA kit from Beyotime Biotechnology company. The competitor probe was non-biotinylated TAATTAATTAATTAATTAATTAATTAATTAAT sequence.

## 3. Results

### 3.1. Genome-Wide Identification and Evolutionary Analysis of Plant HD-Zip IV Subfamily Members

**Figure 1:**
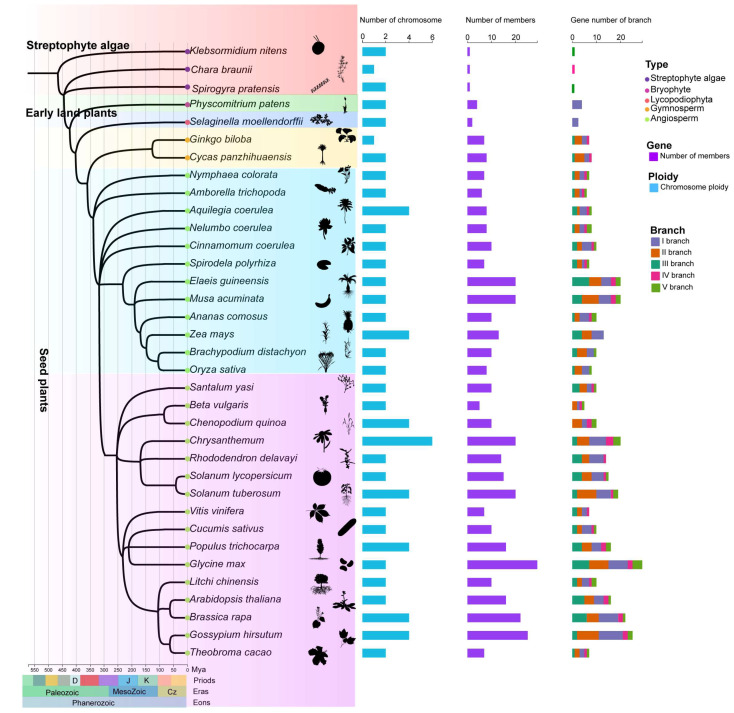
Time-calibrated phylogeny and HD-Zip IV subfamily gene count of 35 species. The figure includes a geologic timescale representing the chronological system of plant evolution. Time (MYA): indicating millions of years ago.

**Figure 2:**
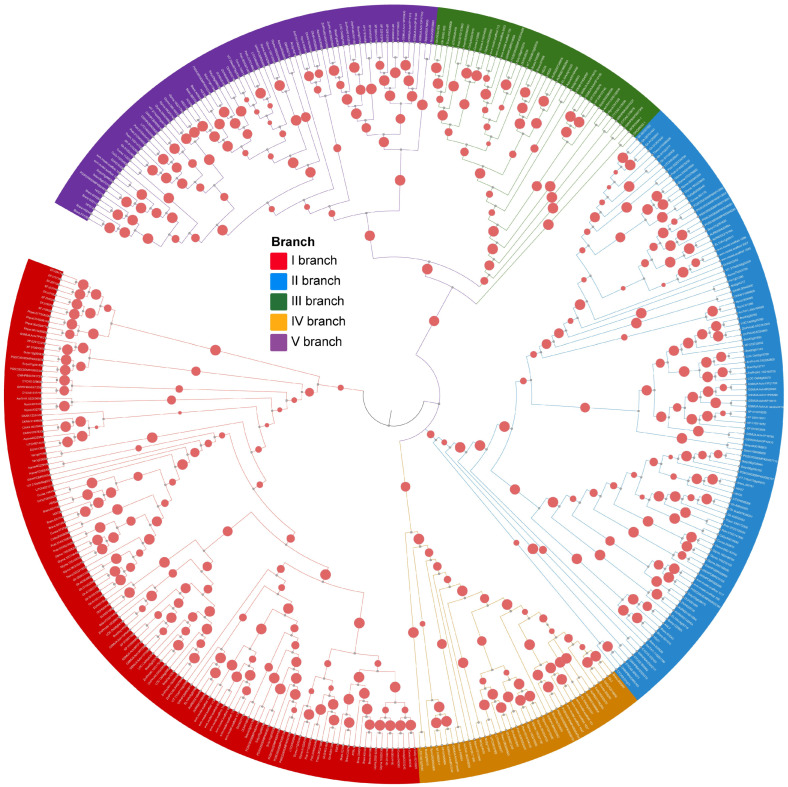
Phylogenetic tree of 393 HD-Zip IV family proteins in plants.

To comprehensively investigate the evolutionary expansion of HD-Zip IV subfamily genes in plants, 35 representative species were selected, encompassing major evolutionary branches from algae to angiosperms. These species include three streptophyte algae, one bryophyte, onelycopodiophyte, two gymnosperms, and twenty-eight angiosperms from various orders, spanning over hundreds of millions of years in plant evolution (Figure 1; Table S1). A total of 393 HD-Zip IV subfamily members were identified across these 35 species and classified into five evolutionary branches, consistent with the phylogenetic analysis of HD-Zip IV subfamily members in *Arabidopsis* (Figure 2; Figure S1). In the streptophyte algaes, only one HD-Zip IV subfamily members was identified in each of *chlorophyta* species studied: *Klebsormidium nitens*, *Chara braunii* and *Spirogyra pratensis*. In contrast, four members were detected in the bryophytes specie *Physcomitrium patens*, and eight were identified in the lycophyte species *Selaginella moellendorffii*. Among seed plants, the number of HD-Zip IV subfamily members varied significantly, ranging from 5 to 29 (Figure 2; Figure S2). From early land plants to seed plants, the HD-Zip IV subfamilies exhibited expansion of members and branches; however, the degree of expansion varied markedly across species, with no apparent correlation to their evolutionary position or development complexity. Furthermore, an analysis of the distribution of 393 HD-Zip IV subfamily sequences across branches revealed that, in the streptophyte algaes and early land plants, genes were restricted to one branch. In contrast, the HD-Zip IV subfamily of seed plants expanded into five branch (Figure S2). Gymnosperms and certain monocots lacked HD-Zip IV subfamily members in branch IV and branch V. This analysis suggests that the HD-Zip IV subfamily show an evolutionary expansion in geological timescales.

### 3.2. Genomic Identification and Evolutionary Expansion Analysis of the HD-Zip IV Subfamily in Non-Seed Plants

**Figure 3:**
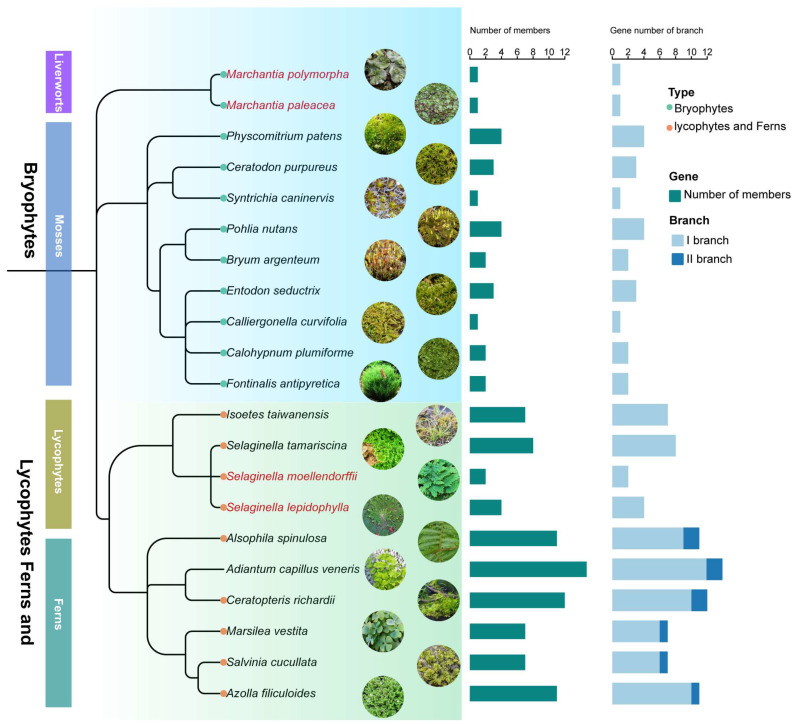
Time-calibrated phylogeny and HD-Zip IV subfamily gene count of 11 bryophytes and 10 lycophytes and ferns.

To explore the evolutionary expansion of the HD-Zip IV subfamily in early-diverging land plants, genomic identification of HD-Zip IV subfamily genes was conducted in 11 bryophytes and 10 lycophytes and ferns (Figure 3; Table S2). A total of 23 sequences were identified in bryophytes and 83 in lycophytes and ferns, resulting in 106 sequences overall (Figure S3). Fewer members were generally observed in bryophytes, which exhibited strong evolutionary conservation and limited gene expansion (Figure 3; Figure S3). In lycophytes and ferns, the number of HD-Zip IV subfamily gene exhibited a rapid expansion. Within the four species of lycophytes, the number of HD-Zip IV subfamily members ranged from 2 to 8, progressively increasing to 14 members in *Adiantum capillus*, and gradually diverged into new evolutionary branch (Figure 3; Figure S3). This trend suggests that the expansion of HD-Zip IV subfamily genes became more pronounced as plants became more terrestrial.

### 3.3. Functional Divergence Analysis of Plant HD-Zip IV Subfamily Proteins

**Figure 4:**
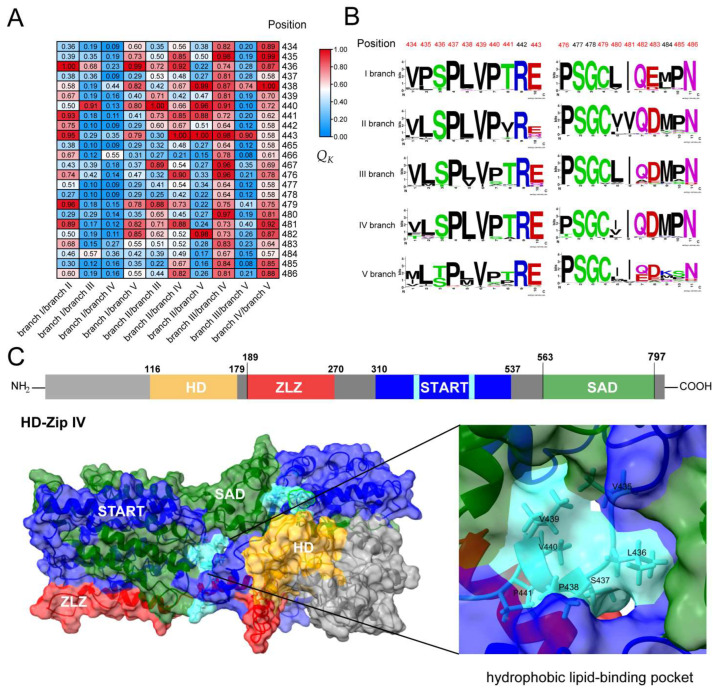
Functional divergence analysis of HD-Zip IV subfamily proteins in plants. (**A**) Heatmap showing divergence sites with *Q_K_* > 0.8 among the five evolutionary branches of HD-Zip IV subfamily. (**B**) Conservation analysis of amino acid divergence across evolutionary branches. (**C**) Protein structure diagram of HD-Zip IV subfamily members and three-dimensional structure representation of HD-Zip IV subfamily proteins. The divergence sites are localized to the lipid-binding pocket, the structure is shown in the light-blue section.

Throughout the evolutionary history of plant terrestrialization, the HD-Zip IV subfamily has served as a critical transcriptional regulator, orchestrating plant hormone signaling, which in turn affects epidermal cell fate and properties. While functional redundancy exists among evolutionary branches, distinct roles, and functional divergence are evident. This is supported by the characterization of the five evolutionary branches of the *Arabidopsis* HD-Zip IV subfamily (Figure S1). To explore the genetic and evolutionary basis of functional divergence among different evolutionary branches of the HD-Zip IV subfamily, type I and type II divergence analyses were conducted among the five branches using DIVERGE 4 software. The analysis revealed positive *θ*_1_ values for all branch pairs except the branch I/branch IV pair (*θ*_1_ ± S.E. < 0; LRT < 1). Several amino acid sites showed posterior probabilities of *Q_k_* > 0.8 (Table 1). Functional divergence sites were identified across branches as follows: 5 (I/II), 1 (I/III), 5 (I/V), 3 (II/III), 7 (II/IV), 5 (II/V), 13 (III/IV), 1 (III/V), and 9 (IV/V) (Figure 4A,B; Table S3). These sites were concentrated at the bottom and sides of the hydrophobic lipid-binding pocket in the START domain of HD-Zip IV subfamily transcription factors (Figure 4C). Type II analyses showed negative *θ*_2_ ± S.E. values among all branches, suggesting that functional divergence is driven by amino acid evolution rate in the START domain rather than changes in physicochemical properties.

**Figure 5:**
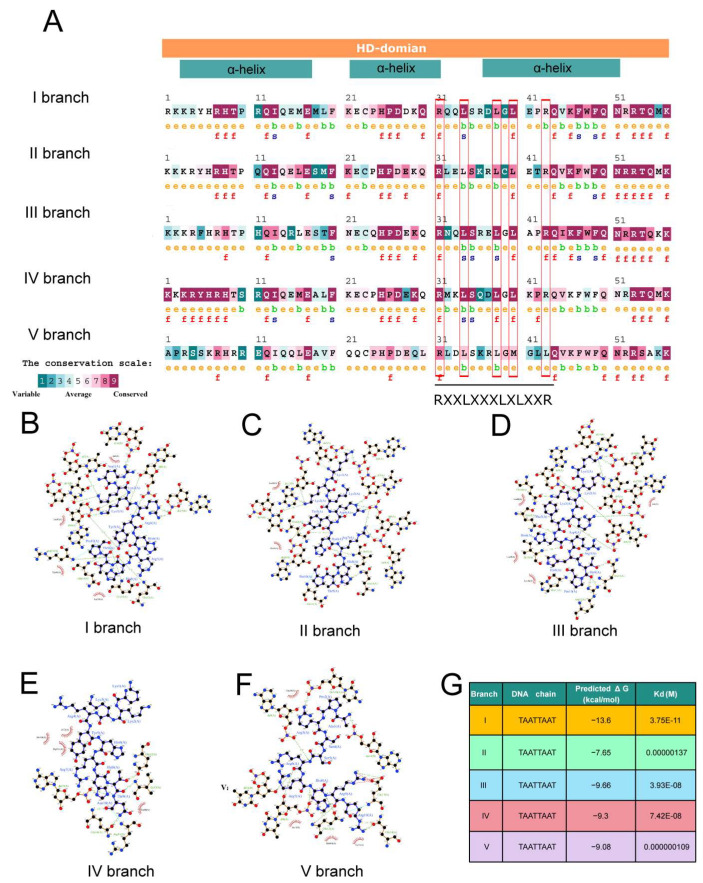
Amino acid variations and DNA binding affinity in the HD. (**A**) Conservation analysis of HD across different branches of the HD-Zip IV subfamily. The conservation grades range from 1 (variable) to 9 (highly conserved). (**B**–**F**) Docking visualizations of the HD of five branches with L1-box core sequences. (**G**) Predicted protein-DNA binding affinities the HD of five branches with L1-box core sequences.

**Figure 6:**
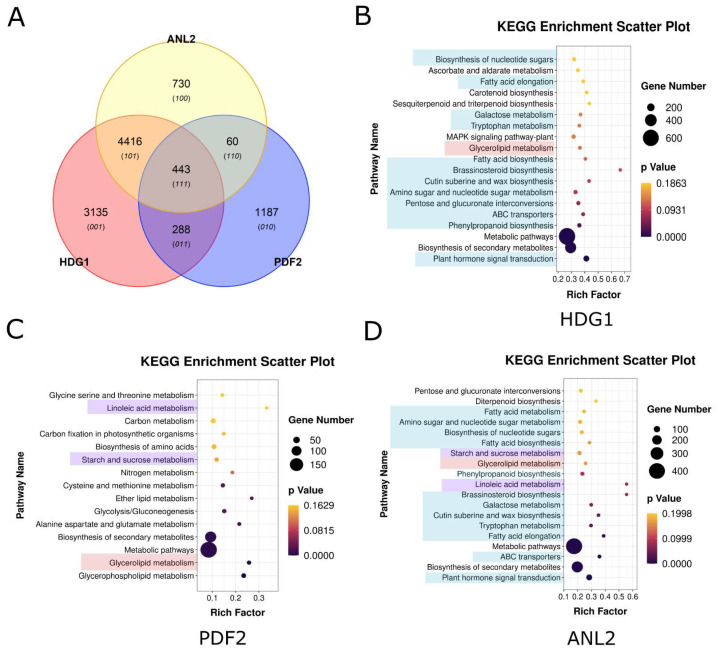
Target gene analysis of HD-Zip IV subfamily transcription factors in *A. thaliana*. (**A**) Venn diagram illustrating target gene overlap among HD-Zip IV subfamily members. (**B**–**D**) KEGG pathway enrichment analyses for HDG1, PDF2 and ANL2 regulatory target genes. The highlighted components represent the KEGG pathways that are shared among the target gene sets of the three transcription factors.

As lipid/steroid binding transcription factors, HD-Zip IV subfamily proteins play a critical role in lipid–ligand interactions through the START domain. Additionally, the HD, responsible for DNA binding and regulation, contributes significantly to functional differentiation. Conservation analysis using conSurf software identified amino acid variations in the HD across branches, in particular, a leucine-rich motif RXXLXXXLXLXXR in HD (Figure 5A). Docking analyses using HDOCK and subsequent affinity evaluations with LigPlot and PDA-Pred revealed significant differences in the hydrophobic interactions and binding stability between HD and the L1 box core sequence (TAATTAAT) across branches (Figure 5B–F). For example, the HD of branch I and the TAATTAAT motif exhibited stronger hydrophobic interaction and higher binding free energy (ΔG), indicating more stable DNA binding (Figure 5B,G). In contrast, the HD of branch II demonstrated weaker interactions, with a ΔG of −7.65 kcal/mol, suggesting reduced binding stability (Figure 5C,G). The hydrophobic interactions of branches III, IV, and V were more concentrated, with intermediate binding stability between branches I and II (Figure 5D–G). These differences in DNA binding affinity and binding specificity likely contribute to variations in the regulation of downstream target genes, driving functional divergence among branches. DAP-seq analysis of *Arabidopsis* HD-Zip IV subfamily members supports this conclusion (Figure 6). For instance, branch I protein PDF2, with high binding specificity to the L1 box, regulates 1980 target genes. In contrast, branch II proteins ANL2 and HDG1, which exhibit weaker binding specificity to the L1-box, regulate 5146 and 7551 genes, respectively, with significant overlap (4416 co-regulated genes) and consistent functional pathways (Figure 6A–D).

### 3.4. Adaptive Evolution and Convergent Selection Analysis of Early Evolutionary Branches

Darwinian positive selection plays a pivotal role in shaping the adaptive evolution of plant gene families. In this study, we examined the selective pressure influencing the evolutionary trajectory of the HD-Zip IV subfamily I branch during plant evolution. Using easycodeML software, we analyzed adaptive evolution in HD-Zip IV subfamily branch I genes in plants. In the site-specific model analysis, the ω (omega) value of the M0 model was 0.70164, indicating that the HD-Zip IV subfamily has been predominantly subjected to purifying selection. Comparisons of models M3 and M2a with models M0 and M1a revealed no positively selected amino acid sites. However, the comparison of model M8 with model M7 identified 47 positively selected sites, mainly concentrated within the START domain (Table 2; Table S3). These findings suggest that certain amino acid sites within the START domain of branch I in the HD-Zip IV subfamily have undergone strong positive selection. To assess whether selective pressures influenced the evolutionary transition of HD-Zip IV subfamily genes from early land plant to seed plant, a branch-site model analysis was conducted. Designating early land plant HD-Zip IV subfamily genes from branch I as the foreground branch, the comparison of branch-site model A with the null model (LRT, *p* = 0.0998871617) revealed no significant positive selection sites between early land plants and seed plant HD-Zip IV subfamily genes (Table 2). Thus, branch I genes of the HD-Zip IV subfamily are largely constrained by purifying selection across conserved domains such as HD, Zipper–Loop–Zipper’s (ZLZ) START adjacent domain (SAD). In contrast, specific sites within the START itself show signatures of adaptive evolution.

**Figure 7:**
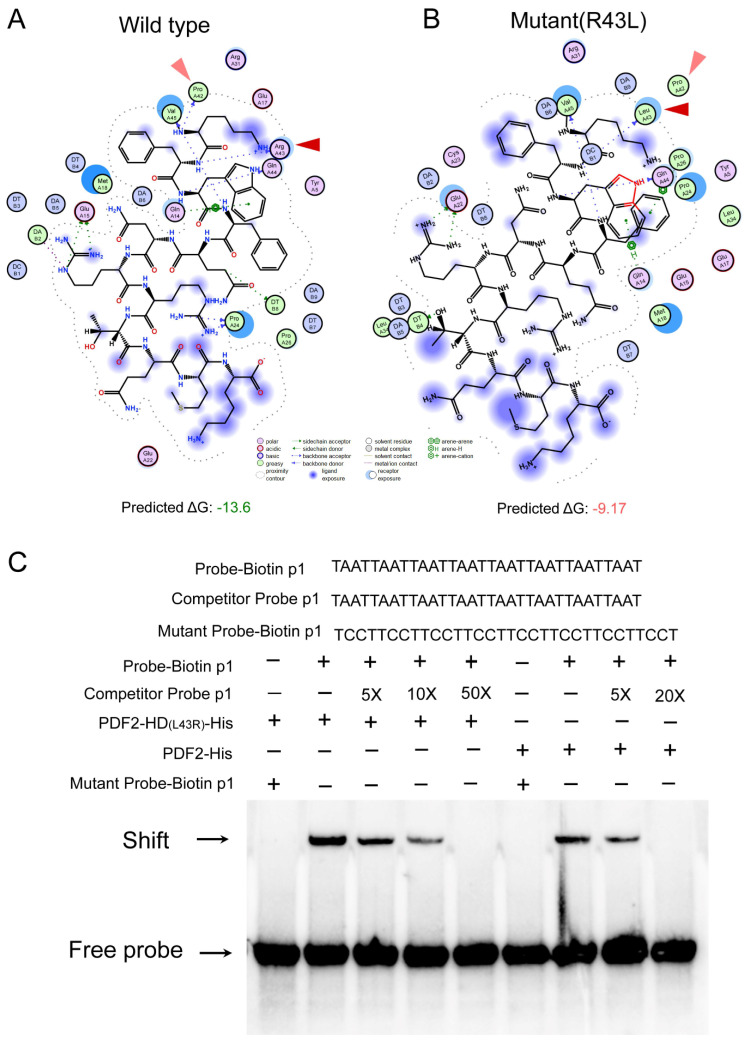
Convergent evolution in HD-Zip IV subfamily I branch. (**A**) Docking visualization of the wild-type HD with L1-box motifs. The red and light-red triangular arrows are used to indicate the forces exerted on the specific residues at positions 42 and 43 in HD domian during the molecular interaction with the L1-box motif. (**B**) Docking visualization of the R43L mutant HD with L1-box motifs. The red and light-red triangular arrows are used to indicate the forces exerted on the specific residues at positions 42 and 43 in HD domian during the molecular interaction with the L1-box motif. (**C**) Electrophoretic mobility shift assays were performed to examine the binding of PDF2-his and its mutant PDF2-HD_(L43R)_-his proteins with the biotin-labeled L1-box core motif. Shift represent gel shift band, free probe refers to the unbound probe that has not formed a complex with the target protein.

A convergent evolution analysis was also performed for HD-Zip IV subfamily branch I genes. Convergent evolution sites were detected in the HD and SAD between early land plants and seed plants. In the HD, the following amino acid mutations were observed: at position 10, non-polar fatty acid residues (L/P/A/V) mutated to the polar amino acid Q; at position 32, neutral residues M/Q mutated to the basic amino acid K; and at position 43, the basic amino acid R mutated to the non-polar residue L (Figure S4). The R43L mutation decreased the binding free energy (ΔG) between the HD and the L1-box core sequence (TAATTAAT) from −13.6 kcal/mol to −9.17 kcal/mol, reducing binding stability (Figure 7A,B). Moreover, EMSA assays confirmed that the L43R mutation in the HD of PDF2 (a branch I member of Arabidopsis HD-Zip IV subfamily) significantly enhanced its binding capacity to the tandem repeats of the L1 box core motif (Figure 7C) These findings imply that relative to those early land plants, a binding affinity- and specificity-weakened mutation to the L1 box core motif has become widespread in seed plants during evolution. In the SAD, seven convergent evolution sites were identified: Q496E, T499V, N501S, N535D, E538R, A643D, and S707D. These mutations may significantly alter the protein properties (Figure S5). These findings indicate that HD-Zip IV subfamily branch I genes exhibit molecular-level convergent evolution in both the HD and SAD domains, contributing to functional optimization and adaptive modifications across diverse plant lineages.

## 4. Discussion

### 4.1. The Evolutionary History of the HD-Zip IV Subfamily Provides Insights into Plant Terrestrialization

Gene families emerge from ancestral genes through mechanisms such as duplication and transposition. The transition from single to multiple members and the functional diversification of gene families reflect major evolutionary processes, including natural selection, genome duplication, and genetic drift [38]. In this study, we found that the first duplication or expansion of the HD-Zip IV subfamily occurred after the Marchantiales (Figure 3), and the members of HD-Zip IV subfamily gradually expand from early land plants to seed plants, especially after selaginella moellendorffii (Figure 1). At these HD-Zip IV gene family expansion nodes, Guan et al. confirmed that Marchantiales, an early-diverging lineage of land plants, represents a pivotal stage in the evolutionary transition of plants to terrestrial environments [39,40]. During this period, plants began developing central vascular bundles and enhancing cuticle formation, driven by alternating dry and wet conditions and long-term symbiotic interactions with fungi [41,42]. Furthermore, the early vascular plant *Selaginella moellendorffii* is characterized by the development of foundational terrestrial adaptations, including vascular system, stomata and a cuticle [43,44]. In summary, these findings suggest that the expansion of the HD-Zip IV subfamily is intricately tied to plant terrestrialization, playing a key role in the emergence of terrestrial adaptive traits.

From another perspective, our findings reveal that the evolution of HD-Zip IV subfamily maintained relatively conservation and only minimal gene expansion in bryophytes (Figure S3), and these was a rapid expansion of HD-Zip IV subfamily members and new evolutionary branches emerged majority of lycophyte and fern species (Figure 3). This result may be related to the WGD events or possibly horizontal gene transfer of lycophytes and ferns, which occurred about 30 million years ago [45,46]. Conversely, the HD-Zip IV subfamily did not expand in *Selaginella* species, which lack WGD events [47]. For instance, *Selaginella moellendorffii* and *Selaginella tamariscina* possess only two and four HD-Zip IV subfamily members, respectively (Figure 3). Such variation is also observed in seed plants. Beta vulgaris (sugar beet) has only half the number of HD-Zip IV subfamily members identified in *Chenopodium quinoa*, despite both being *Amaranthaceae* species (Figure 1). *Beta vulgaris* has also experienced fewer genome duplication events than other species in its lineage. Moreover, *Chenopodium quinoa* shows greater resilience to extreme conditions than *Beta vulgaris*, and overexpressing the HD-Zip IV subfamily gene *EsHdzip1* exhibited increased drought resistance in tobacco [48,49,50]. These observations suggest that the evolution of the HD-Zip IV subfamily is not only synchronized with plant terrestrialization on the evolutionary timescale but also serves as a key player and molecular record of pivotal evolutionary events in plants. This provides invaluable insights into evolutionary processes spanning hundreds of millions of years. These observations suggest that the expansion of the HD-Zip IV subfamily was closely linked to genome duplication events and that it was an important contributor to the evolution of terrestrial adaptation in plants.

### 4.2. Site Variations in the HD and START Domains Is a Key Driver for Functional Diversification in the HD-Zip IV Subfamily

A comparative analysis of amino acid conservation within the HD-Zip IV subfamily revealed that divergence sites are primarily concentrated in the HD and START domains, specifically, in the leucine-rich region (spanning residues 31–43) of the HD (Figure 5A). Among these sites, variations at positions 31, 38, and 40 have been shown to influence the DNA binding specificity of HD-Zip IV subfamily transcription factor [51]. Meanwhile, our EMSA assays on the divergence site variation at site 43 in the HD domain demonstrated that this mutation in a conserved residue significantly alters the binding affinity of HD-Zip IV transcription factors for the L1-box motif (Figure 7C). Thus, these divergence sites ultimately led to significant differences in the predicted binding affinity for the L1-box motif among different subfamily lineages (Figure 5G). This functional divergence, in turn, resulted in starkly different regulatory target sets between branch I member PDF2 and branch II members HDG1/ANL2 of the *Arabidopsis* HD-Zip IV subfamily (Figure 6). In all, these findings highlight significant functional differentiation among HD-Zip IV family branches, driven primarily by amino acid variations in the HD domain.

Similarly, variations in the START domain are likely pivotal in the evolution and functional diversification of the HD-Zip IV subfamily. This study identified numerous branch-specific variations within the START domain, primarily concentrated in the hydrophobic lipid-binding pocket (Figure 4C). These variations can influence the START domain’s ability to bind lipid molecules, thereby affecting lipid-binding affinity and responses to environmental stress [52,53]. Furthermore, deletion or mutation of the START can also influence the regulatory activity of HD-Zip IV subfamily transcription factors by altering their dimerization and protein stability [54]. Together, these variations in the HD and START synergistically drive the functional diversification of the HD-Zip IV subfamily.

### 4.3. Balance Strategy in the Adaptive Evolution of the HD-Zip IV Subfamily

In biological evolution, purifying selection and positive selection are two opposing forces that collectively drive adaptive evolution. This study reveals that both purifying and positive selection simultaneously influence the evolution of the HD-Zip IV subfamily branch I, targeting distinct domains (Table 2). A total of 47 positive selection sites in branch I are concentrated in the START domain, and the abundance of positively selected sites suggests that the START domain has undergone strong positive selection during plant evolution (Table S3). Other gene families also show an abundance of positive selection sites similar to what we observed. In the NBS-LRR disease resistance gene family, Mariana et al. identified widespread positive selection within the LRR domain, reflecting the ongoing co-evolution between host plants and pathogens [55]. In the citrus NBS gene family, 18–24 positively selected sites were identified per branch, with the accumulation of these mutations accelerating functional divergence [56]. Similarly, in the GRAS gene family, evolutionary analysis revealed 11 strongly positively selected sites within α-helical domains in branches I and V, contributing to the evolution of conserved motifs and functional specialization [57]. Although some of the 47 positively selected sites found in the HD-Zip IV subfamily I branch may represent compensatory mutations with limited evolutionary impact, our findings underscore the substantial adaptive pressures acting on the START during evolution. These pressures have driven the evolutionary diversification of HD-Zip IV subfamilies, enabling their adaptation to increasingly complex terrestrial environments. In contrast, other domains of the HD-Zip IV subfamily, the HD, namely, ZLZ, and SAD are predominantly shaped by purifying selection (Figure 4C). This ensures the functional stability of these domains, which play critical roles in maintaining conserved physiological functions. This strategy, balancing differential positive and purifying selection across various domains, is commonly observed in the evolution of numerous animal and plant species. Such functional variation resulting from this balance grants organisms an enhanced adaptive capacity, allowing them to succeed in diverse and changing habitats [58,59]. Therefore, the balance between functional robustness and relaxed constraints may confer greater functional flexibility for the HD-Zip IV subfamily to respond to challenging land environments.

## Figures and Tables

**Table 1:** Functional divergence analysis of HD-Zip IV subfamily branches.

Group	Type I Divergence					Type II Divergence	
	*θ*1 ± S.E.	LRT	*p*	Q_k_ > 0.8	Q_k_ > 0.9	*θ*2 ± S.E.	Q_k_ > 0.8
branch I/branch II	0.5912 ± 0.127528958305202	21.49073289	<0.01	5	4	0.503329127 ± 0.486213472	0
branch I/branch III	0.2728 ± 0.142546928692003	3.662460952	<0.01	1	1	−1.126225006	0
branch I/branch IV	0.1864 ± 0.234102844227785	0.633984202	<0.01	0	0	−0.137818643	0
branch I/branch V	0.5328 ± 0.159572502908097	11.14839424	<0.01	5	1	−0.773091545	0
branch II/branch III	0.5312 ± 0.141214757037623	14.14998457	<0.01	3	1	0.509979579 ± 0.479703046	0
branch II/branch IV	0.636 ± 0.166487202456239	14.59326671	<0.01	7	3	0.439594831 ± 0.379396093	0
branch II/branch V	0.4304 ± 0.0837889901146688	26.38582108	<0.01	5	4	0.367849079 ± 0.427968197	9
branch III/branch IV	0.8016 ± 0.213136792132244	14.14485719	<0.01	13	6	0.252762068 ± 0.403554606	0
branch III/branch V	0.2552 ± 0.137036387360895	3.468083443	<0.01	1	0	−0.298762699	0
branch IV/branch V	0.736 ± 0.17754372635251	17.18481919	<0.01	9	3	0.132169223 ± 0.345319597	1

**Table 2:** Positive selection sites in HD-Zip IV subfamily branch I genes.

Model	Ln L	Estimates of Parameters					Model Compared	LRT *p*-Value	Positive Sites
M3	−205,593.959399	*p*:	0.00208	0.37473	0.62319		M0 vs. M3	0.000000000	None
		ω:	0.00038	0.20788	0.51335				
M0	−206,514.653780	ω0:	0.70164						Not Allowed
M2a	−206,034.403370	*p*:	0.00207	0.50427	0.49366		M1a vs. M2a	0.000000000	None
		ω:	0.00162	1.00000	1.77705				
M1a	−226,322.298272	*p*:	0.71915	0.28085					Not Allowed
		ω:	0.00000	1.00000					
M8	−206,267.262537	*p*0 = 0.00207	*p* = 0.03339	q = 2.21096			M7 vs. M8	0.000000000	See Table S4
		(*p*1 = 0.99793)	ω= 1.00000						
M7	−207,009.677476	*p* = 0.00501		q = 0.00536					Not Allowed
M8a	−214,523.123117	*p*0 = 0.98520	*p* = 0.00500	q = 1.76218			M8a vs. M8	0.000000000	Not Allowed
		(*p*1 = 0.01480)	ω= 1.00000						
Branch site-Model A	−206,266.163925	Site class	0	1	2a	2b	Model A vs. Model A null	0.998871617	None
		f	0.00202	0.97319	0.00005	0.02474			
		ω0	0.00140	1.00000	0.00140	1.00000			
		ω1	0.00140	1.00000	3.72117	3.72117			
Branch site-Model A null	−206,266.163926	1							Not Allowed

## Data Availability

No new data were generated for this study. All data are included in the manuscript.

## Supplementary Materials

The following supporting information can be downloaded at https://www.mdpi.com/article/10.3390/genes16111348/s1, Figure S1. Phylogenetic tree of HD-Zip IV family proteins in *A. thaliana*. Figure S2. The number of members in the five evolutionary branches of the HD-Zip IV family across different species. Figure S3. Phylogenetic tree of HD-Zip IV family proteins in 21 plant species, namely, 11 bryophytes and 10 pteridophytes. Figure S4. Convergent evolution sites in HD of HD-Zip IV subfamily I branch. Figure S5. Convergent evolutionary sites in the HD-Zip IV SAD across plant species. Table S1. Names and genus information of the 35 species. Table S2. Detailed information on the names and genus of the 21 species. Table S3. Positive selective site identified using the M8 model.
