# Supplementary material for: Evolutionary Diversification and Adaptive Evolution Analysis of the Plant HD-Zip IV Subfamily
Source: Genes (Basel). 2025 Nov 8;16(11):1348. doi: 10.3390/genes16111348 (PMC12652440; doi:10.3390/genes16111348)
Supplement: Supplementary file 1 [file genes-16-01348-s001.zip › genes-3972008-supplementary.pdf]

Supplementary Figure

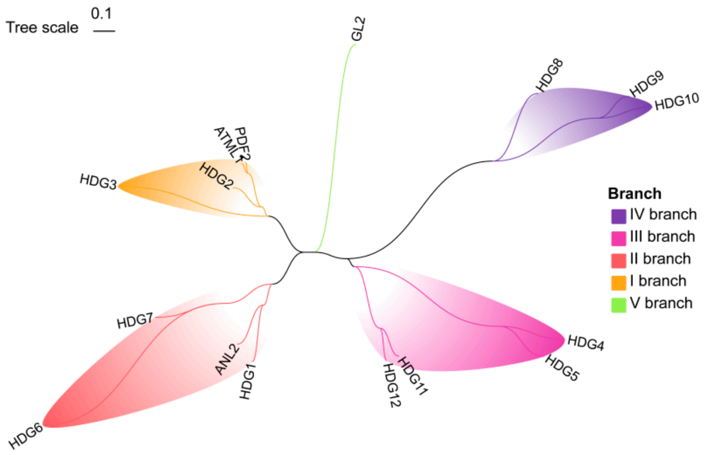

Figure S1. Phylogenetic tree of HD-Zip IV family proteins in *Arabidopsis thaliana*.

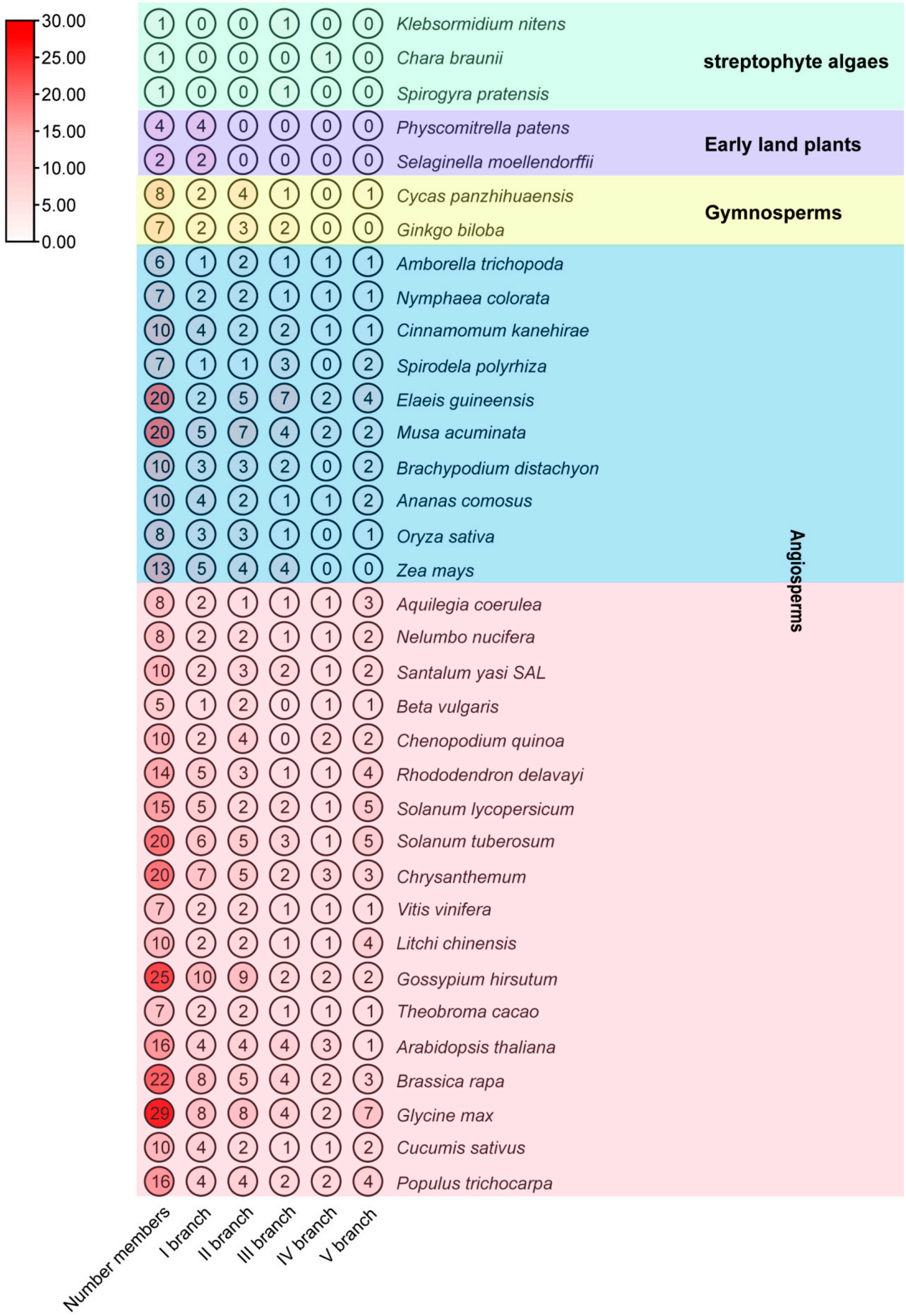

Figure S2. The number of members in the five evolutionary branches of the HD-Zip IV family across different species.

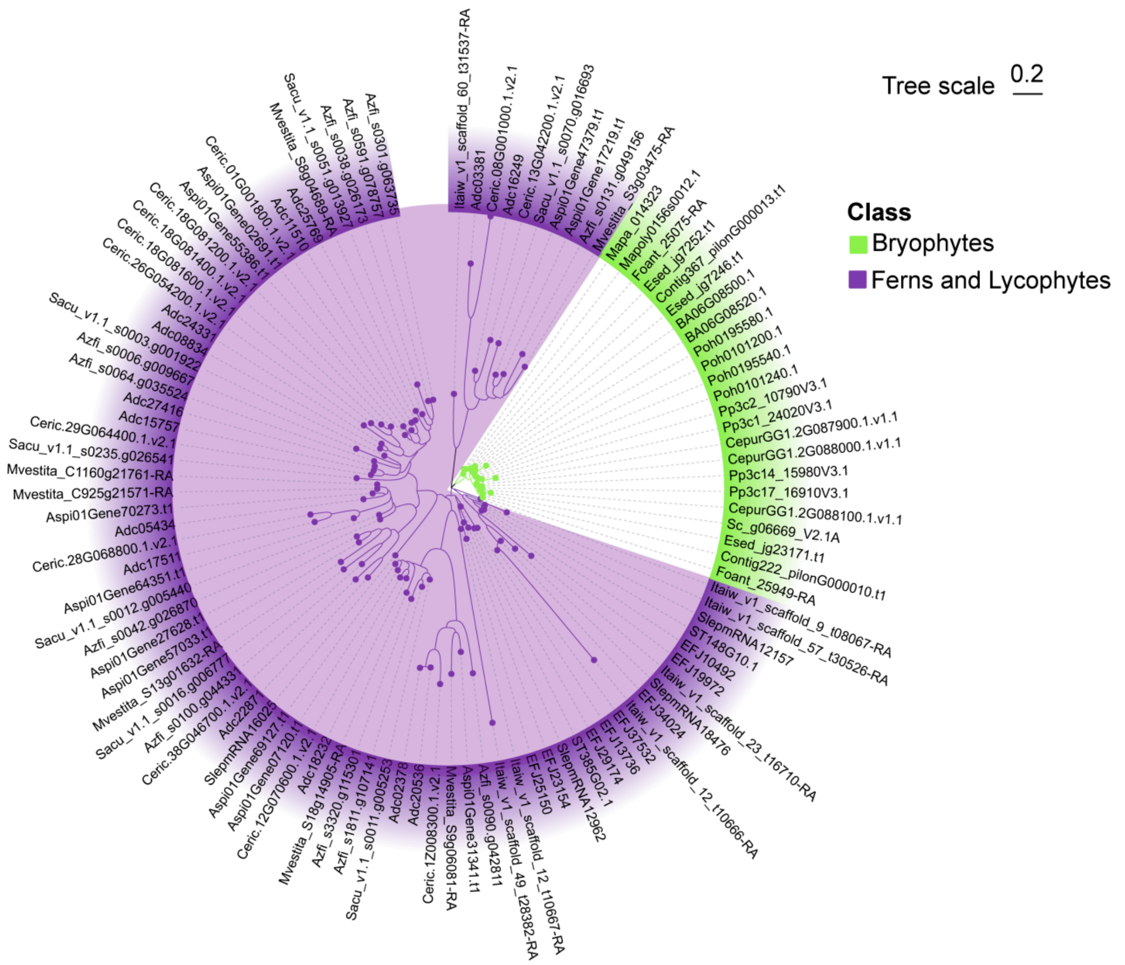

Figure S3. Phylogenetic tree of HD-Zip IV family proteins in 21 plant species, namely, 11 bryophytes and 10 pteridophytes.

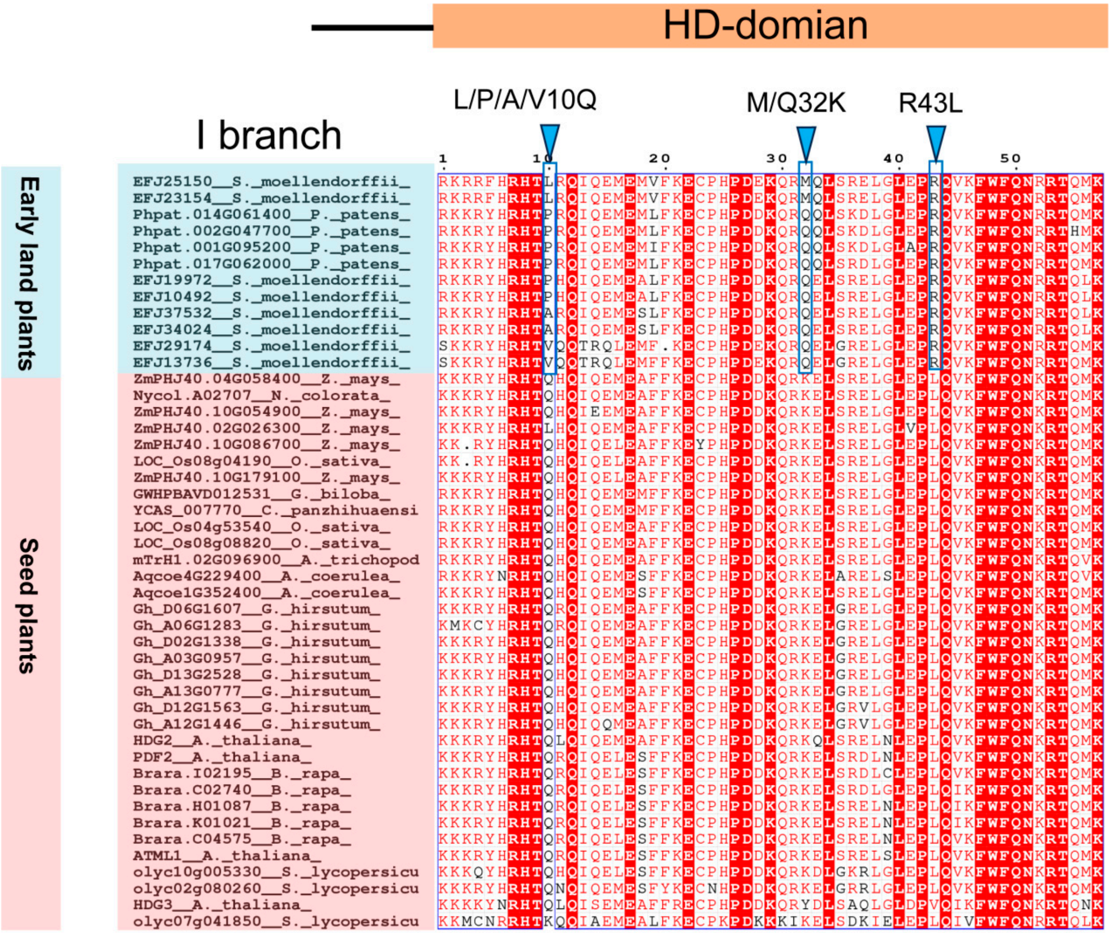

Figure S4. Convergent evolution sites in HD of HD-Zip IV subfamily I branch.

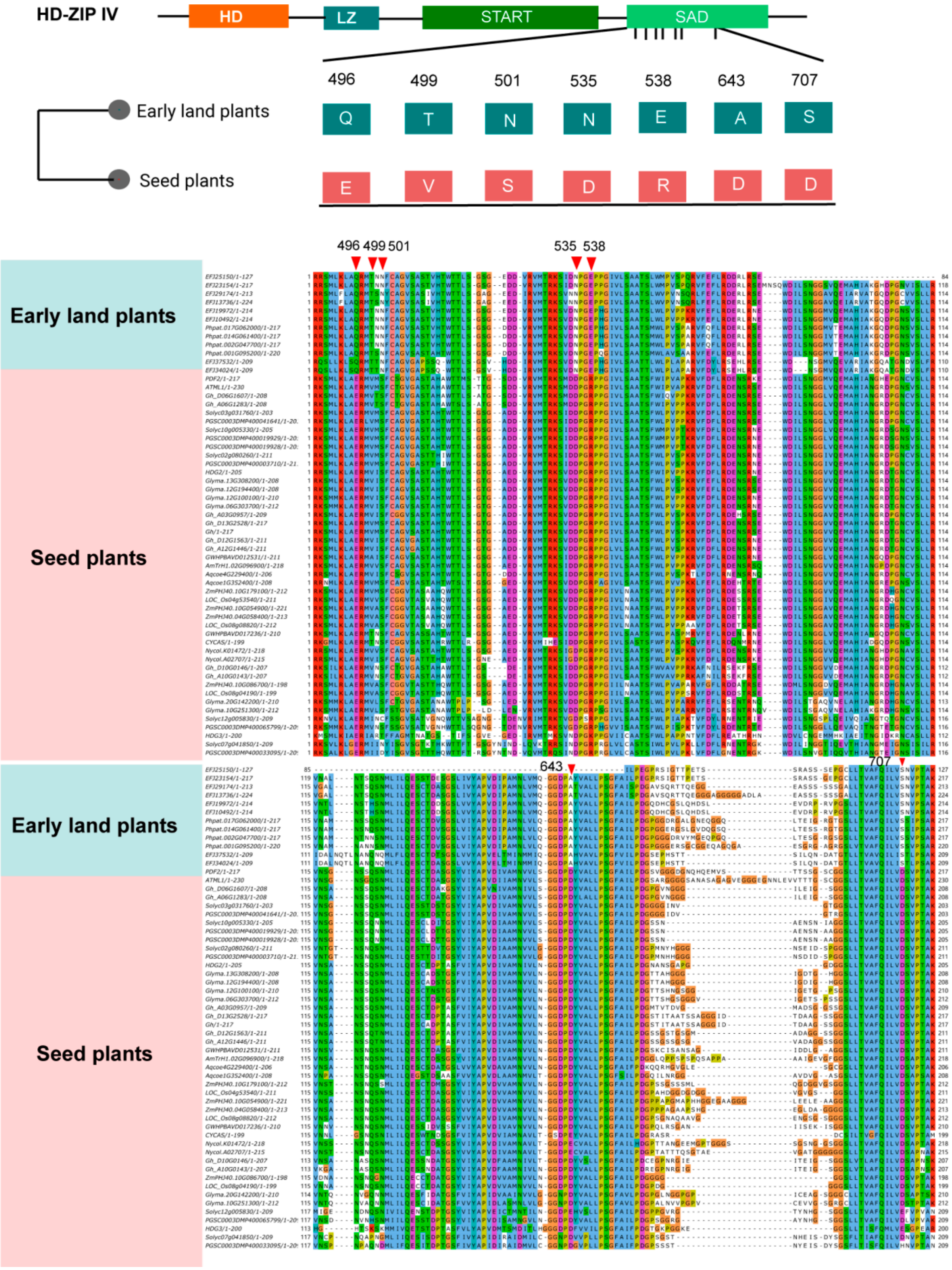

Figure S5. Convergent evolutionary sites in the HD-Zip IV SAD domain across plant species.

Supplementary Table

Table S1. Names and genus information of the 35 species.

Number	Species name	NCBI taxonomy ID	Orders	Number	Species name	NCBI taxonomy ID	Orders
1	*Klebsormidium nitens*	105231	*Zygnematales*	19	*Nelumbo nucifera*	4432	*Proteales*
2	*Chara braunii*	69332	*Charales*	20	*Santalum yasi SAL*	453089	*Santalales*
3	*Spirogyra pratensis*	332123	*Charales*	21	*Beta vulgaris*	161934	*Caryophyllales*
4	*Physcomitrella patens*	3218	*Funariales*	22	*Chenopodium quinoa*	63459	*Caryophyllales*
5	*Selaginella moellendorffii*	88036	*Selaginellales*	23	*Rhododendron delavayi*	321363	*Ericales*
6	*Cycas panzhihuaensis*	123604	*Cycadales*	24	*Solanum lycopersicum*	4081	*Solanales*
7	*Ginkgo biloba*	3311	*Ginkgopsida*	25	*Solanum tuberosum*	4113	*Solanales*
8	*Amborella trichopoda*	13333	*Amborellales*	26	*chrysanthemum*	13422	*Asterales*
9	*Nymphaea colorata*	210225	*Nymphaeales*	27	*Vitis vinifera*	29760	*Vitales*
10	*Cinnamomum kanehirae*	337451	*Laurales*	28	*Litchi chinensis*	151069	*Sapindales*
11	*Spirodela polyrhiza*	29656	*Alismatales*	29	*Gossypium hirsutum*	3635	*Malvales*
12	*Elaeis guineensis*	51953	*Arecales*	30	*Theobroma cacao*	3641	*Malvales*
13	*Musa acuminata*	4641	*Zingiberales*	31	*Arabidopsis thaliana*	3702	*Brassicales*
14	*Brachypodium distachyon*	15368	*Poales*	32	*Brassica rapa*	3711	*Brassicales*
15	*Ananas comosus*	4615	*Poales*	33	*Glycine max*	3847	*Fabales*
16	*Oryza sativa*	4530	*Poales*	34	*Cucumis sativus*	3659	*Cucurbitales*
17	*Zea mays*	4577	*Cyperales*	35	*Populus trichocarpa*	3694	*Malpighiales*
18	*Aquilegia coerulea*	218851	*Ranales*				

Table S2. Detailed information on the names and genus of the 21 species.

Number	Species name	NCBI taxonomy ID	Orders	Number	Species name	NCBI taxonomy ID	Orders
1	*Calliergonella curvifolia*	2029240	*Hypnobryales*	12	*Selaginella lepidophylla*	59777	*Selaginellales*
2	*Pohlia nutans*	140635	*Bryales*	13	*Selaginella moellendorffii*	88036	*Selaginellales*
3	*Syntrichia caninervis*	200751	*Pottiales*	14	*Selaginella tamariscina*	137178	*Selaginellales*
4	*Marchantia paleacea*	56867	*Marchantiales*	15	*Isoetes taiwanensis*	99432	*Isoetales*
5	*Ceratodon purpureus*	3225	*Dicranales*	16	*Adiantum capillus-veneris*	13818	*Filicales*
6	*Fontinalis antipyretica*	67435	*Hypnobryales*	17	*Alsophila spinulosa*	204586	*Cyatheales*
7	*Calohypnum plumiforme*	98943	*Hypnobryales*	18	*Azolla filiculoides*	84609	*Salviniales*
8	*Marchantia polymorpha*	3197	*Marchantiales*	19	*Ceratopteris richardii*	49495	*Polypodiales*
9	*Entodon seductrix*	105143	*Hypnobryales*	20	*Marsilea vestita*	59764	*Salviniales*
10	*Bryum argenteum*	37413	*Eubryales*	21	*Salvinia cucullata*	32188	*Salviniales*
11	*Physcomitrium patens*	3218	*Eubryales*				

Table S3. Positive selective site identified using the M8 model.

Number	Positive sites	Pr(w>1) p-value	Number	Positive sites	Pr(w>1) p-value
1	268 D	0.967*	25	433 H	0.988*
2	286 Y	0.967*	26	436 V	0.976*
3	287 F	0.986*	27	437 D	0.999**
4	294 I	0.985*	28	440 S	0.960*
5	320 I	0.958*	29	441 V	0.999**
6	351 A	0.967*	30	443 N	0.968*
7	352 G	0.969*	31	445 Y	0.983*
8	356 G	0.992**	32	446 K	0.958*
9	375 R	0.995**	33	447 P	0.977*
10	379 F	0.974*	34	451 T	0.959*
11	381 R	0.964*	35	453 L	0.983*
12	382 Y	0.974*	36	456 G	0.959*
13	389 G	0.982*	37	463 T	0.955*
14	392 A	0.958*	38	464 L	0.975*
15	404 S	0.971*	39	465 D	0.988*
16	406 I	0.954*	40	468 C	0.982*
17	417 L	0.954*	41	471 L	0.987*
18	419 Q	0.954*	42	473 S	0.981*
19	420 E	0.970*	43	476 A	0.991**
20	421 L	0.978*	44	478 N	0.970*
21	425 Y	0.993**	45	481 A	0.968*
22	430 W	0.974*	46	482 C	0.968*
23	431 V	0.986*	47	484 L	0.987*
24	432 E	0.961*			

Note: Positively selected sites (*: P>95%; **: P>99%).
